# Statistical Optimization of the Sol–Gel Electrospinning Process Conditions for Preparation of Polyamide 6/66 Nanofiber Bundles

**DOI:** 10.1186/s11671-018-2644-9

**Published:** 2018-08-08

**Authors:** Edgar Franco, Rosmery Dussán, Maribel Amú, Diana Navia

**Affiliations:** 1 Architecture, Urbanism, and Esthetics Research Group, Faculty of Architecture, Art and Design, University of San Buenaventura, Cali, Colombia; 2 Faculty of Engineering, University of San Buenaventura, Cali, Colombia

**Keywords:** Sol–gel electrospinning, Polyamide 6/66, Nanofiber bundles, Scanning electron microscope, Differential scanning calorimeter

## Abstract

Polymeric nanofibers are widely studied in the textile industry since with them, it is possible to get a great variety of functionalities. In this paper, polyamide 6/66 (PA 6/66) solutions at different concentrations (12, 17, and 22% wt.) were made, to get nanofibers through the basic electrospinning process which were characterized by scanning electron microscope (SEM) and productivity. Afterwards, nanofiber bundles were produced using the electrospinning sol–gel process, which were characterized by SEM and tensile test. From the results of statistical optimization based on one-way analysis of variance (ANOVA) with post hoc Tukey HSD, it was found that nanofiber bundles with higher productivity (1.39 ± 0.15 mg/min), draw ratio (9.0 ± 1.2), and tensile strength (29.64 ± 7.40 MPa) were obtained with a 17% concentration. Finally, a thermal characterization through differential scanning calorimetry (DSC) was done, finding evidence of a *T*_g_ and *T*_m_ reduction in the nanofibers in relation to PA 6/66 pellets and nanofiber bundles.

## Background

Nylon is a polymer classified as polyamide that was discovered by Wallace Hume Carothers in 1934; it is produced as fiber and plastic depending on the processing conditions [1]. Commercially, there are different kinds of nylon, nylon 6, nylon 66, nylon 6, 10, etc., having in common the amide functional group (–CO-NH-) [2]. This polymer is used to produce blown films and monofilaments through spinning processes, and it can be copolymerized. Such is the case of nylon 6/66 which is produced to lower melting temperature compared to nylon 6. In the last few years, nylon has been used in multiple applications such as women stockings, parachutes, zippers, fishing lines, bridal veils, carpets, musical strings, and rope [3].

Conventional filament and nylon thread transformation processes are wet spinning, dry spinning, and gel spinning and allow to manufacture between 20- and 400-μm diameter filaments [4]. These processes, which are carried out from polymeric solutions, are dependent on the concentration, since their variation affects the stretching ratio and consequently the mechanical properties of the fibers [5].

The electrospinning process [6] is used, even at a nanometer scale, to get nylon fibers of a smaller diameter [7]. It also allows to make polymeric nanofibers with polarities, porosities, and adjustable diameters that can additionally be adapted to a wide variety of sizes and shapes. Besides, by using this technique, it is possible to control the properties, functionality, and composition of the nanofibers through the polymer concentration and electrospinning parameters [8]. Ramkrisna et al. [9] affirm that the morphological result of the electrospinning process presents a high concentration dependence, as a similar way with conventional spinning processes [5]; since a higher concentration leads to a higher viscosity in the polymer solution, for this reason, the study of this article evaluates the concentration of the polymer as unique variable in a unifactorial design. This is important to evaluate the possibility of carried out on an industrial scale.

Polymeric nanofibers obtained through electrospinning can be used in many fields in industry: scaffolds, sensors, filters, membranes, batteries, protective clothing, wound dressing, and catalyst [10]. In the textile area, nanofibers are used to get specific functionalities such as self-cleaning fabrics, virus and bacteria repellent, temperature control, sensors, and filters [11]. Also, some other textile applications have been reported as antibacterial clothing [12], wound dressings [13], and protective clothing [14] thanks to their chemical properties and mechanical strength.

**Fig. 1:**
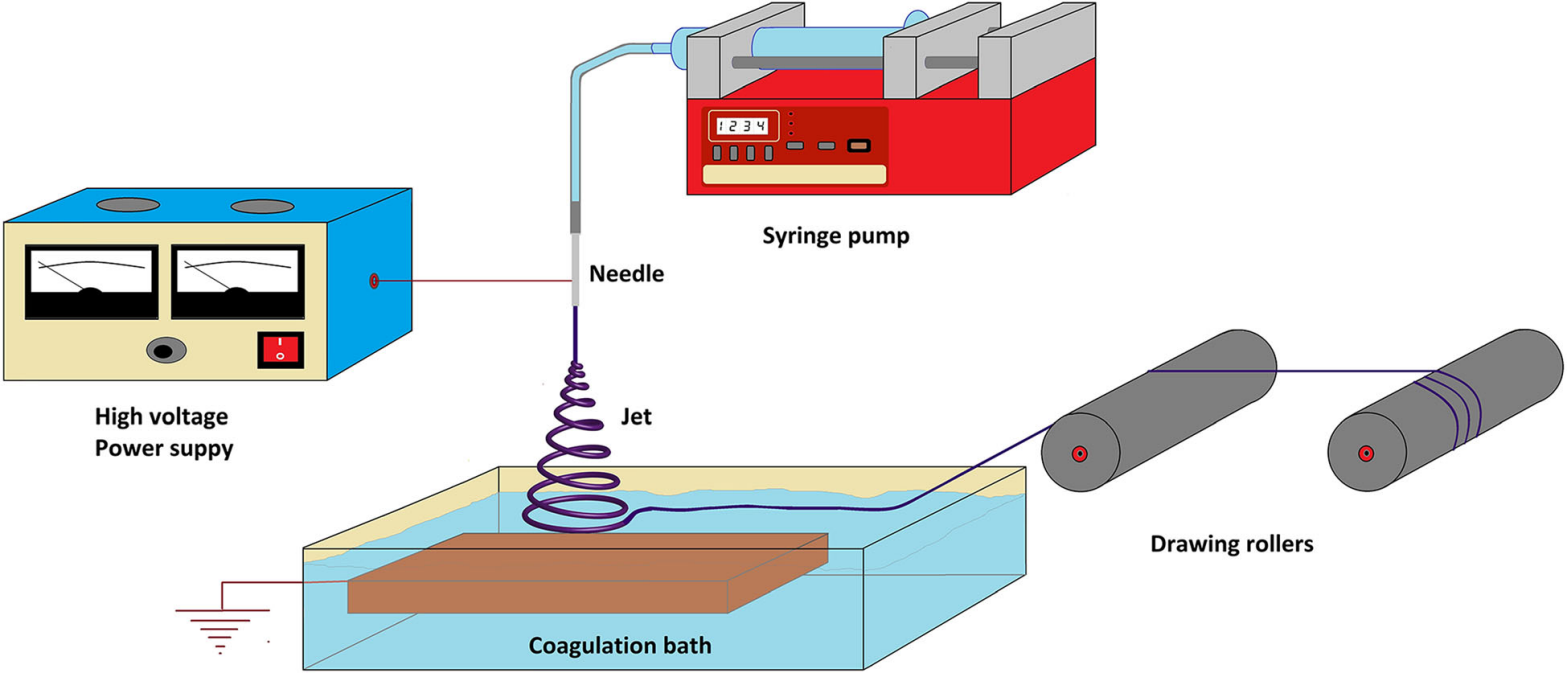
Electrospinning sol–gel process

Some other researches, related to the electrospinning process, adapted it to a coagulation bath and tensile cylinders to develop the electrospinning sol–gel process (see Fig. 1). With the latter, polyvinyl alcohol (PVA) nanofiber bundles were characterized, produced, and applied as a secondary reinforcement in ultra-fine Portland cement pastes, reducing retraction and width in fissures at early ages of setting [15]. In a paper published by Wu et al. [16], they prepared and characterized aggregated nanofibers of polyamide 6/66, through the electrospinning process, using two collector rolls that rotated with a speed of 300 r.p.m. They stated that the fibers obtained have a wide range of applications in tissue scaffolds, composite reinforcement and ultrasensitive sensors [17]. By using a similar methodology here, we present the results and statistical optimization using ANOVA with post hoc Tukey HSD test of the morphological, productivity mechanical, and thermal characterization of polyamide 6/66 (PA 6/66) nanofiber bundles obtained through such process where the effect of the polymer concentration on the final properties of the resulting fibers for their subsequent use in the textile field was studied.

## Methods

### Materials

PA 6/66, reference Ultramid C40 L, Basf brand was used. As solvent, a mixture of formic acid and acetic acid was used, and distilled water as the coagulation bath.

### Preparation of Polyamide Solutions

Solutions were prepared at different concentrations in weight 12%wt., 17% wt., and 22% wt., using a mixture of formic acid and acetic acid as solvent in a mass relation of 4:1 [18], at room temperature and continuous stirring.

### Basic Electrospinning Process

In all the process carried out with the solutions at different concentrations, a voltage of 27.5 kV was used, supplied by a Gamma High Voltage Research Inc. Model E30 equipment connected to a metal needle placed at 12 cm from the collector. The flow of the solution was controlled by a syringe pump from Braintree Scientific Syringe Pump Brand Inc. which was set between 0.3 and 1 ml/h.

### Electrospinning Sol–Gel Process

This process was carried out using a coagulation bath of distilled water and a tensile cylinder’s system with speed control. We got nanofiber bundles of PA 6/66 at three concentrations (12, 17, and 22%), following a unifactorial design completely random with three replicas and a significance level of *α* = 0.05. Studied variables were productivity in the deposit of nanofibers, draw ratio, and nanofiber bundle tensile strength. It is important to note that concentrations less than 12% by weight were not evaluated, since in preliminary tests this condition did not allow the formation of electrospinning nanofibers; in the same way, no concentrations greater than 22% by weight were evaluated, because the resulting viscosity was very high, hindering agitation in the preparation of the solution and subsequent flow in the electrospinning process. Additionally, only extreme concentrations and the midpoint were investigated.

### Characterization Techniques

For the basic electrospinning process, for each concentration, the productivity (mg/min) in the deposit of electrospun nanofiber mats of PA 6/66 was determined. Afterwards, these were morphologically characterized through scanning electron microscope (SEM).

For the electrospinning sol–gel process, after adjusting process variables (voltage, flow, needle–collector distance), to get a stable and continuous electrospun jet, the draw ratio of the process was determined, then the obtained PA 6/66 nanofiber bundles were characterized through SEM and tensile test. Finally, the process optimal condition was characterized through differential scanning calorimetry (DSC).

#### SEM

The samples were coated in gold in a vacuum coating machine [Denton Vacuum Desk IV] for about 200 s. At the end, they were deposited in the sample holder of scanning electron microscope (JEOL JSM 6490 LV, Japan), equipped with tungsten filament. Afterwards, we induced a 30 Pa vacuum in the chamber to generate electrons, to scan and to get images. Then, with the image software, average nanofiber diameters were measured.

#### Tensile Test

Three hundred threads of nanofiber bundles were tested with a testing machine (EZ-Test L, Shimadzu, Japan) at a trial speed of 30 mm/min and a reference length of 50 mm according to the ASTM D3822 standards.

#### DSC

To determine phase transitions, the differential scanning calorimetry (DSC) technique was used following ASTM D3418-08 standard applied to the polymeric material analysis. We employed a differential scanning calorimeter (DSC) (TA Instruments, Q20, USA) with 5-mg samples that were deposited in hermetically sealed aluminum crucibles and subjected to two consecutive heating cycles from 25 to 250 °C at a 10 °C/min speed with 5 min isotherms between each cycle. The TA Universal Analyzer® Software, adapted to the equipment, allowed to get the thermograms in order to determine the glass-transition temperatures and material fusion.

## Results and Discussion

### Productivity of the Basic Electrospinning Process

**Fig. 2:**
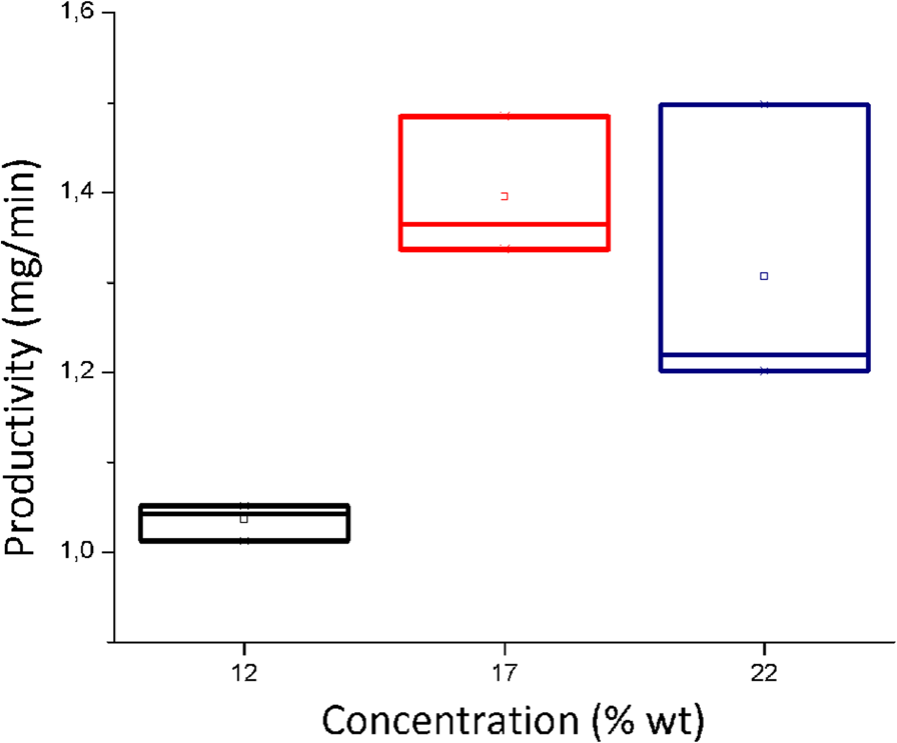
Productivity of the basic electrospinning process

Figure 2 shows productivity results (mg/min) in the nanofiber deposit during the basic electrospinning process at different concentrations. For this variable, the ANOVA generated a *p* value of 0.015. This indicates that at least a median is different. Then, the post hoc Tukey test was applied and it indicated that for this variable, the productivity value averages at concentrations of 17 and 22% are equivalent among each other but higher than the one obtained at 12% concentration.

### Morphology/Morphological Characterization of PA 6/66 Nanofibers

**Fig. 3:**
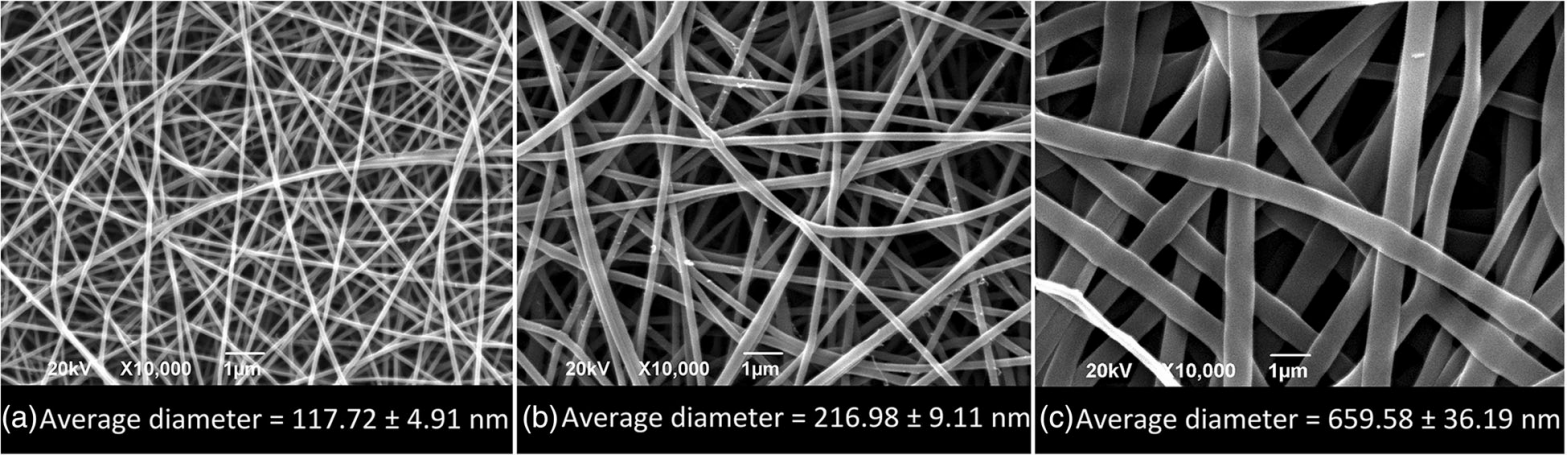
SEM images of PA 6/66 electrospun nanofibers at different concentrations. **a** 12% wt., **b** 17% wt., and **c** 22% wt

By using the basic electrospinning process, PA 6/66 nanofibers at different concentrations were manufactured. The results proved that by increasing the concentration of the polymer solution, the nanofiber diameter increased such as observed in SEM micrographies in Fig. 3. This is due to the fact that increasing the concentration has a rheological thickening effect on the solution [19] which makes it difficult to reduce diameters because of the viscosity rise. This behavior coincides with the reported one by Guerrini et al. [20], who electrospun PA 6/66 nanofibers with different molecular weights. Additionally, it was determined that the average diameters of nanofibers with a 17% concentration increased in approximately 85% compared to nanofibers obtained with a 12% concentration and a 204% for the nanofibers obtained with a 22% concentration compared to those at a 17%.

### Draw Ratio in the Electrospinning Sol–Gel Process

**Fig. 4:**
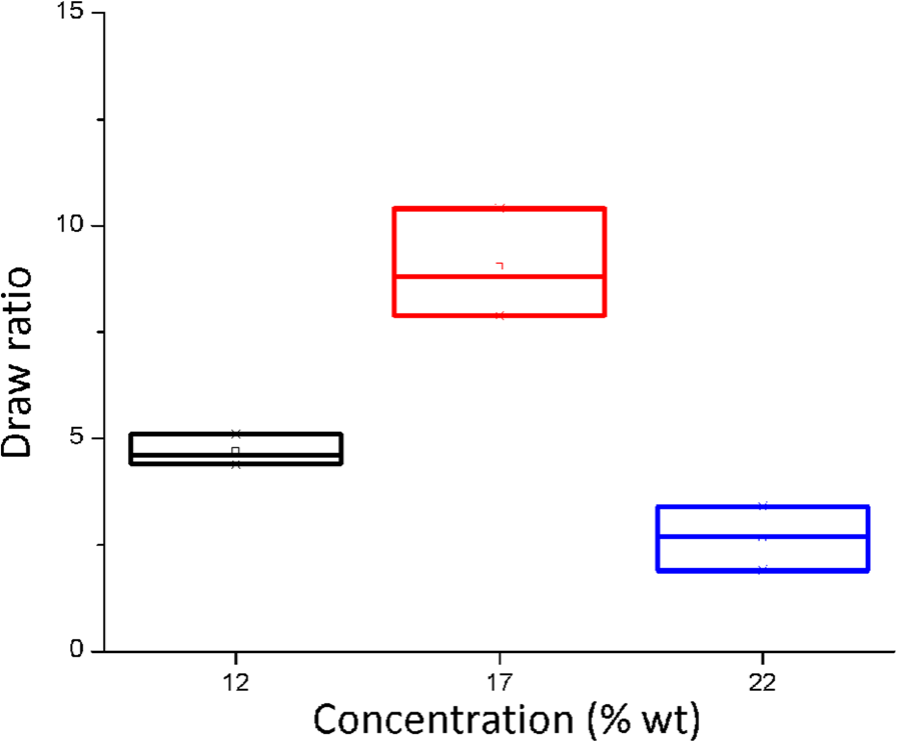
Draw ratio of the electrospinning sol–gel process

Figure 4 shows the draw ratio results measured during the electrospinning sol–gel process at different concentrations. For this variable, the ANOVA generated a *p* value of 0.000 that indicates that at least a median is different. Then, the post hoc Tukey test was applied indicating that for this variable, the draw ratio median obtained at a 17% concentration is higher than the resulting one from 12 and 22% concentrations, which are equivalent among each other.

### PA 6/66 Nanofiber Bundle Morphology/Morphological Characterization

**Fig. 5:**
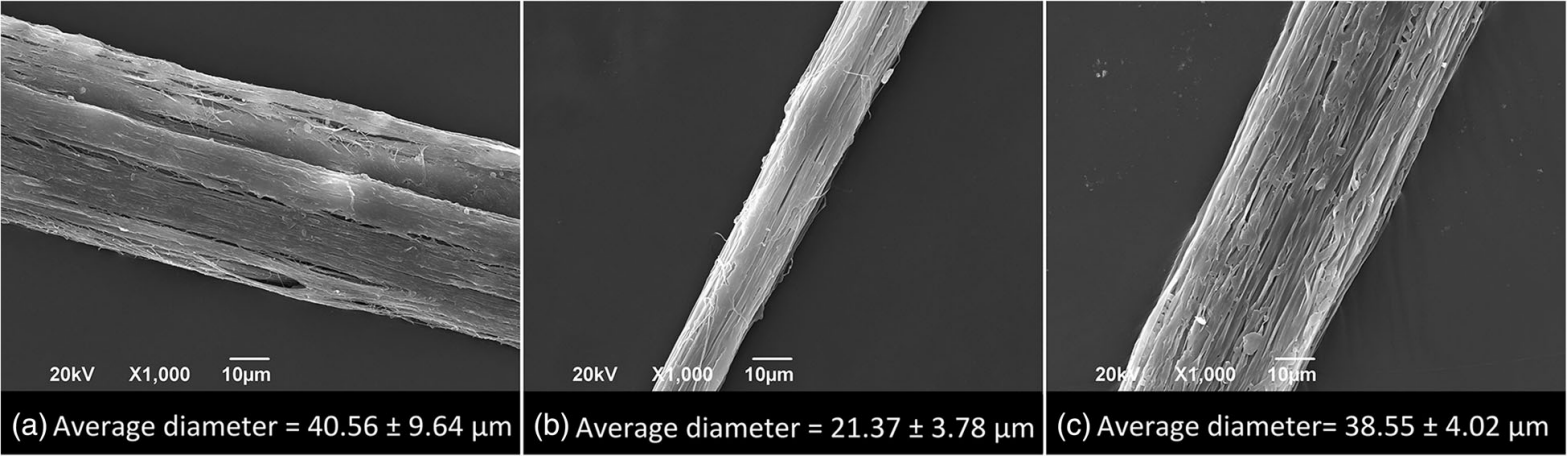
SEM images of polyamide 6/66 nanofiber bundles obtained through electrospinning sol–gel process at different concentrations. **a** 12% wt., **b** 17% wt., and **c** 22% wt.

By using the electrospinning sol–gel process, polyamide nanofiber bundles were made at different concentrations in the solution. After measuring their diameters, it was found that the smaller average one was reached at 17% concentration as observed in SEM micrographies in Fig. 5. The resulting nanofiber bundles with a 17% concentration, reached diameters of almost the half of the ones obtained with 12 and 22% concentrations respectively. This is due to the fact that the process was carried out at a fibers higher recollection speed and a greater draw ratio.

### Nanofiber Bundle Tensile Strength

**Fig. 6:**
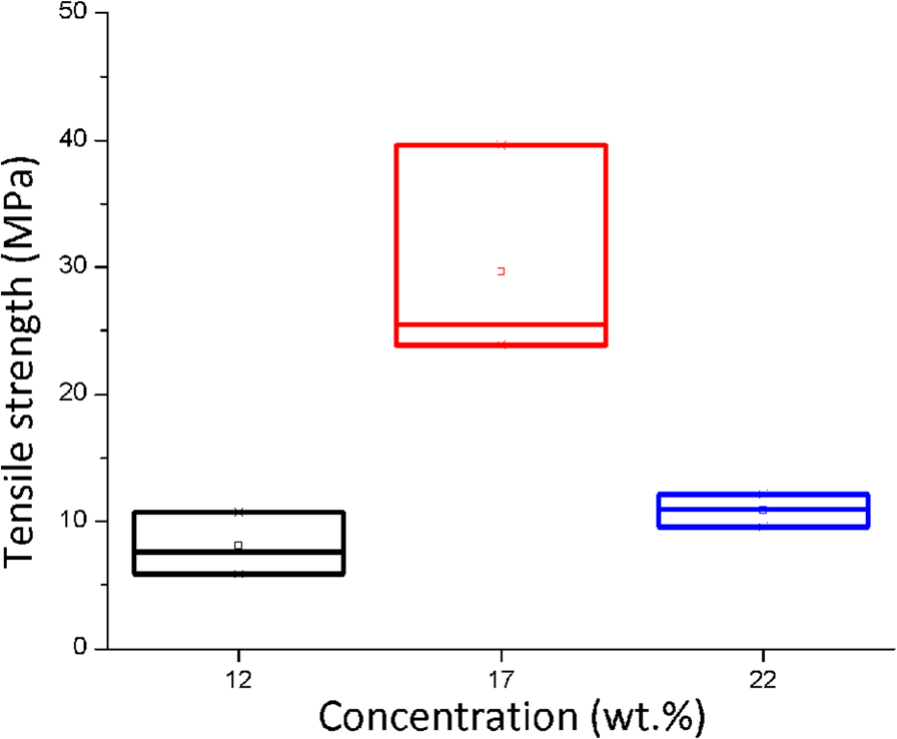
Tensile strength of polyamide 6/66 nanofiber bundles at different concentrations

Figure 6 presents the boxes diagram of the tensile strength, measured over the nanofiber bundles obtained during the electrospinning sol–gel process at different concentrations. For this variable, the ANOVA generated a *p* value of 0.005 indicating that at least a median is different. Afterwards, the post hoc Tukey test was applied showing that for this variable the tensile strength average of nanofiber bundles obtained at a 17% concentration is higher than the result from the 12 and 22% concentrations which are equivalent among each other.

Additionally, the average result of tensile strength obtained with the 17% nylon (29.64 MPa) concentration was similar to the one reported by Wu et al. [16] in their research presenting results of 66 polyamide nanofibers firstly electrospun and then bent with strength values near 30 MPa.

### Optimal Process Condition

**Fig. 7:**
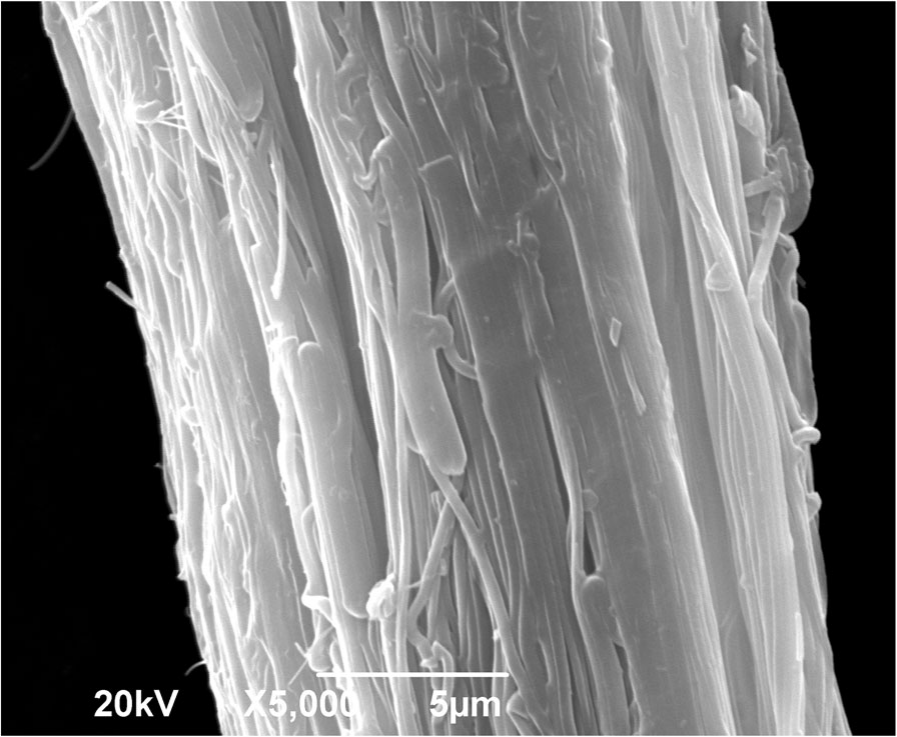
SEM image of the surface of PA 6/66 nanofiber bundles

Previous results show that developing the electrospinning sol–gel process from a PA 6/66 solution at a 17%wt. concentration allows to produce nanofiber bundles with a higher productivity, draw ratio, and tensile strength. Additionally, Fig. 7 shows, more closely, the nanofiber bundles making it possible to observe a rise in the superficial roughness. This is important if what is intended is to use these fibers as composite material reinforcement, due to the fact that they allow a better mechanical fixation to the matrix if compared to conventional synthetic fibers that usually have a smooth surface. Additionally, these fibers have a high ratio of area to volume and high aspect ratio, which gives them potential in applications in the design of clothing, filters, and nanocomposites [21].

### DSC Thermal Analysis of the Optimal Condition Nanofiber Bundles

**Fig. 8:**
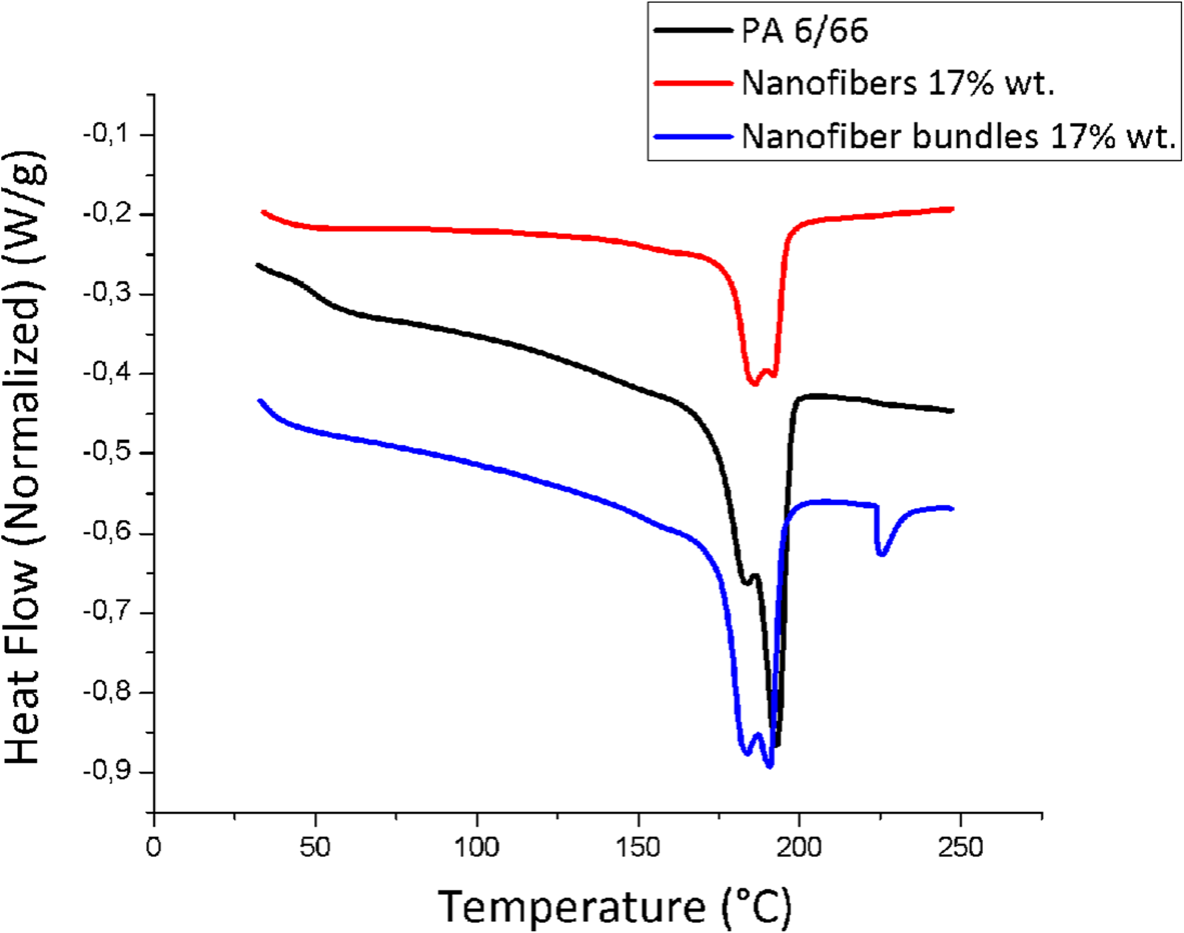
Resulting optimal condition nanofiber bundle thermograms obtained through DSC

From the optimal condition at 17% concentration, we carried out a thermal analysis of each of the transformation stages since the material is in pellets, it turns into nanofibers and is finally transformed into nanofiber bundles. Figure 8 shows the calorimetry test results obtained through DSC for each stage.

Based on these thermograms, we calculated the glass-transition temperature (*T*_g_), the melting temperature (*T*_m_), the heat of fusion (Δ*H*_m_), and the degree of crystallinity (*X*_c_). These results may be observed in Table 1.

**Table 1:** Thermal parameters obtained by DSC results

Samples	*T*_g_ (°C)	*T*_m_ (°C)	Δ*H*_m_ (J/g)	*X*_c_(%)
PA 6/66	51.33	192.80	74.16	39.45
Nanofibers 17%	35.93	186.28	23.13	12.30
Nanofiber bundles 17%	40.29	190.79	33.47	17.80

*X*_c_ degree of crystallinity obtained by DSC, taken from Δ*H*_m_/Δ*H*_m100%_; Δ*H*_m100%_ heat of fusion for PA6, 100% crystalline, 188 J/g [24]

It may be observed from the *T*_g_ results that nanofibers at a 17% concentration show a higher intermolecular mobility, compared to the nanofiber bundles at the same concentration. The latter is explained because the increased molecular mobility is caused by a rise in the polymer chain space called free volume, which reduces interactions among them. This way, chains with greater mobility require lower temperature for the transition from vitreous solid to rubbery, resulting in lower *T*_g_ values.

The melting temperature value is associated with the required temperature to melt the ordered structures (crystals) into the polymer, and its variation is related to the crystal size. It may be noted that nanofibers at a 17% concentration showed the lowest value in the melting temperature compared with the PA and nanofiber bundles at the same concentration. This indicates that the basic electrospinning process and electrospinning sol–gel reduced the amount of crystalline regions of the polymer in relation to the pellets, turning them into fibril structures [22], which through the spinning and the applied draw ratio, oriented the polymeric chains and showed a recovery in the crystallinity degree of the nanofiber bundles in a 44.71% compared to nanofibers. Finally, the enthalpy of fusion reveals the crystallinity amount in the analyzed polymer [23], and its value is associated with the required energy in the crystalline structure fusion. This proves that nanofibers at a 17% concentration demand less energy to melt than the crystalline structures from the nanofiber bundles and the PA 6/66 pellets.

## Conclusions

6/66 polyamide electrospinning sol–gel process showed a significant increase in the productivity (1.39 ± 0.15 mg/min), draw ratio (9.0 ± 1.2), and tensile strength (29.64 ± 7.40 MPa) for a 17% concentration in weight compared to the other two tested concentrations (12% wt. and 22% wt.). This statistically optimized process condition allowed us to get uniform PA 6/66 nanofiber bundles from a stable and continuous process.

## Abbreviations

DSC: Differential scanning calorimetry
PA 6/66: Polyamide 6/66
PVA: Polyvinyl alcohol
SEM: Scanning electron microscope
